# Supplementary material for: The CXCR4-Dependent LASP1-Ago2 Interaction in Triple-Negative Breast Cancer
Source: Cancers (Basel). 2020 Aug 29;12(9):2455. doi: 10.3390/cancers12092455 (PMC7564666; doi:10.3390/cancers12092455)
Supplement: Supplementary file 1 [file cancers-12-02455-s001.docx]

Supplementary Material: The CXCR4-Dependent LASP1-Ago2 Interaction in Triple-Negative Breast Cancer

Augustus M. C. Tilley, Cory M. Howard, Sangita Sridharan, Boopathi Subramaniyan, Nicole Bearss, Sawsan Alkhalili and Dayanidhi Raman

**Figure S1.** LASP1 Associates with Ago1: (**A**) GST-pulldown assay of Ago1 with 1mg of 231S lysate incubated with 1.5 nmol of each GST-LASP1 domain. Detection of Ago1 was performed by western blotting and detection of LASP1 and its domains was performed by Ponceau Stain (*n* = 3). (**B**) Image of full blot of figure S1A.

**Figure S2.** LASP1 Associates with Ago2 from 231S Lysates: (**A**) Single-mutant GST-pulldown assay for Ago2 with 1mg of 231S lysate incubated with 1.5 nmol of each single-mutant. Detection of Ago2 performed by western blotting and detection of GST-mutants done with Ponceau Stain (*n* = 3). (**B**) Double-mutant GST-pulldown assay for Ago2 with 1 mg of 231S lysate incubated with 1.5 nmol of each double-mutant. Detection of Ago2 performed by western blotting and GST-mutant detection done by Ponceau Stain (*n* = 3). (**C**) Full blot of figure S2A. (**D**) Full blot of figure S2B.


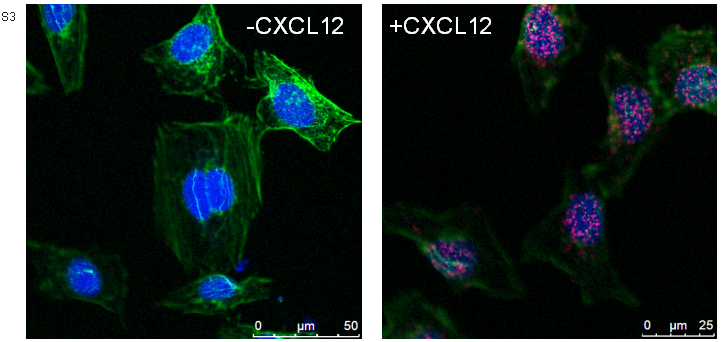


**Figure S3.** LASP1 Interacts with Ago2 in the Nucleus in a CXCR4-Dependent Manner: A PLA utilizing primary antibodies against LASP1 and Ago2 was performed on 231S cells. The red dots represent PLA spots for the LASP1-Ago2 interaction (Scale Bar 25 μm), the blue stain is DRAQ5 for the nucleus (Scale Bar 50 μm), and a green phalloidin stain to outline the cell cytoskeleton. Images were taken by confocal microscopy by taking a series of images at different points on the z-axis. Shown is the image of one z-slice taken on the mid-nuclear plane.

**Figure S4.** Films for Full Blot of Figure 1: (**A**) Image of full blot for figure 1C. (**B**) Image of full blot for figure 1D. (**C**) Image of full blot for figure 1E.

**Figure S5.** Films for Full Blot of Figure 2A.

**Figure S6.** Films for Full Blots of Figure 3: (**A**) Image of full blot for figure 3C. (**B**) Image of full blot for figure 3D. (**C**) Image of full blot for figure 3F. (**D**) Image of full blot for figure 3G.

**Figure S7.** Full blot of figure 5A for LASP1, Ago2 and β-tubulin.

**Figure S8.** Raw Images for Full Blots of Figure 6: (**A**) Full blot for the first half of figure 6A for LASP1 and Ago2. (**B**) Full blot for the first half of figure 6A for β-tubulin. (**C**) Full blot of eIF4G2 and β-tubulin (at high exposure) for the second half of figure 6A. The last four lanes correspond to the bands seen in figure 6A. (**D**) Full blot of vinculin of figure 6B. (**E**) Full blot of β-tubulin and LASP1 (lower exposure) for figure 6B. (**F**) Full blot of β-tubulin, CCR7 and Cyclin D1 at high exposure for the second half of figure 6B.

| 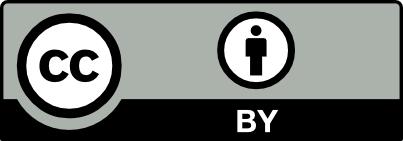 | © 2020 by the authors. Licensee MDPI, Basel, Switzerland. This article is an open access article distributed under the terms and conditions of the Creative Commons Attribution (CC BY) license (http://creativecommons.org/licenses/by/4.0/). |
| --- | --- |
